# Human Glioma Migration and Infiltration Properties as a Target for Personalized Radiation Medicine

**DOI:** 10.3390/cancers10110456

**Published:** 2018-11-20

**Authors:** Michaela Wank, Daniela Schilling, Thomas E. Schmid, Bernhard Meyer, Jens Gempt, Melanie Barz, Jürgen Schlegel, Friederike Liesche, Kerstin A. Kessel, Benedikt Wiestler, Stefanie Bette, Claus Zimmer, Stephanie E. Combs

**Affiliations:** 1 Institute of Innovative Radiotherapy (iRT), Department of Radiation Sciences (DRS), Helmholtz Zentrum München, 85764 Neuherberg, Germany; michaela.wank@helmholtz-muenchen.de (M.W.); Daniela.schilling@tum.de (D.S.); Thomas.Schmid@helmholtz-muenchen.de (T.E.S.); Kerstin.kessel@tum.de (K.A.K.); 2 Department of Radiation Oncology, Technical University of Munich (TUM), Klinikum rechts der Isar, 81675 Munich, Germany; 3 Deutsches Konsortium für Translationale Krebsforschung (DKTK), Partner Site Munich, 81675 Munich, Germany; 4 Department of Neurosurgery, Technical University of Munich (TUM), Klinikum rechts der Isar, 81675 Munich, Germany; Bernhard.Meyer@tum.de (B.M.); Jens.Gempt@tum.de (J.G.); Melanie.Barz@tum.de (M.B.); 5 Department of Neuropathology, Technical University of Munich (TUM), 81675 Munich, Germany; schlegel@tum.de (J.S.); Friederike.Liesche@tum.de (F.L.); 6 Department of Neuroradiology, Klinikum rechts der Isar, Technische Universität München, 81675 Munich, Germany; b.wiestler@tum.de (B.W.); Stefanie.bette@tum.de (S.B.); claus.zimmer@mri.tum.de (C.Z.)

**Keywords:** primary brain tumor, migration and invasion, irradiation, survival, tumor heterogeneity, personalized medicine, brain metastases, radiotherapy, treatment resistance

## Abstract

Gliomas are primary brain tumors that present the majority of malignant adult brain tumors. Gliomas are subdivided into low- and high-grade tumors. Despite extensive research in recent years, the prognosis of malignant glioma patients remains poor. This is caused by naturally highly infiltrative capacities as well as high levels of radio- and chemoresistance. Additionally, it was shown that low linear energy transfer (LET) irradiation enhances migration and invasion of several glioma entities which might counteract today’s treatment concepts. However, this finding is discussed controversially. In the era of personalized medicine, this controversial data might be attributed to the patient-specific heterogeneity that ultimately could be used for treatment. Thus, current developments in glioma therapy should be seen in the context of intrinsic and radiation-enhanced migration and invasion. Due to the natural heterogeneity of glioma cells and different radiation responses, a personalized radiation treatment concept is suggested and alternative radiation concepts are discussed.

## 1. Introduction

Every year, 23,120 tumors of the central nervous system (CNS) are diagnosed in the United States, and 87% of them arise in the brain [1]. Gliomas are primary brain tumors deriving from glial cells and can be separated into low- and high-grade gliomas [2]. However, only 10% are low-grade gliomas, whereas the remaining are classified as high-grade [1]. High-grade gliomas can be subdivided into World Health Organization (WHO) III (anaplastic astrocytoma, anaplastic oligodendroglioma, and anaplastic ependymoma) and WHO IV (glioblastoma) [3]. Glioblastoma multiforme (GBM), which is categorized as grade IV, is the most common and malignant primary brain tumor, with an incidence rate of 3–4/100,000 persons in the United States [4]. As these tumors derive from various brain cell lineages, classification is based on morphological as well as immunohistochemical properties [5]. Despite recent extensive molecular characterization, the standard treatment concept consists of a multimodal treatment regimen that combines surgery with chemo- and radiotherapy [6,7].

Patient survival depends on several factors, but it is generally known that with higher grading, the prognosis gets worse [8]. Despite the considerable effort in glioma research and treatment optimization, the prognosis remains poor [6]. GBM patients have shown a five-year survival rate below 10%, and their median survival is low (4–18 months) [9,10]. As already written in the hallmarks of cancer, proposed by Hanahan and Weinberg, gliomas also exhibit characteristics such as uncontrolled cellular proliferation, diffuse infiltration, resistance to apoptosis, and genomic instability [11]. In addition, intratumoral heterogeneity as well as resistance to chemo- and radiotherapy result in poor prognosis of glioma patients [12]. The poor survival rates are considerably triggered by the aggressive nature of high-grade gliomas and their tendency to diffusely infiltrate into the surrounding tissue. Therefore, it is crucial to find responsible molecular targets that enable a treatment concept to treat this aggressive cancer. Mechanisms that potentially explain the invasive and migratory features as well as radio- and chemoresistance of gliomas have been extensively studied but remain poorly understood.

Ionizing radiation is one of the gold standards in cancer treatment, and it has been proven in many studies that it offers a clear survival benefit for most cancer types [13]. However, it was already revealed in 1991 that the currently applied radiation treatment, using low linear energy transfer (LET) photon irradiation, might favor invasion and migration of gliomas [14]. Since then, this finding has been discussed controversially, and many researchers have shown radiation-enhanced invasion [15,16,17,18]. However, the findings are diverging, and others have proven the opposite [19,20,21]. This might be due to differences in the experimental setup between groups, uncertainties in the assays applied, different dosing and timing regimens of radiations, or other factors related to the experiment. One of the most prominent reasons for the diverging results might also be the substantial heterogeneity between cell lines or patients-specific properties of tumors, respectively. Previously, we have shown substantial heterogeneity in patient-specific tumor cell invasion [15].

As the incidence rate of primary malignant brain tumors is still increasing, while prognosis remains poor, a focus should be set on optimizing current developments in glioma therapy regarding patient-related heterogeneity in light of intrinsic migratory and invasive potential of glioma cells as well as radiation-enhanced migration and invasion.

## 2. Results

### 2.1. Gliomas Exhibit Radiation Resistance

Conventional treatment of high-grade gliomas involves surgical resection followed by radio- and chemotherapy. Despite the advantages in detection, a high-grade glioma patient outcome is still limited to 14 months [22]. That the glioma treatment remains relatively ineffective is attributed to the high radio- and chemoresistance of gliomas as well as to the diffuse infiltration of the neighboring tissue, which has been shown several times. Resistance to radiation could have two possible reasons. First, the tumor cells only receive sublethal doses; second, the cells utilize their repair mechanisms very efficiently [23]. In gliomas, high radioresistance is supposed to be due to intrinsic cellular radioresistance combined with rapid cellular proliferation [24] as well as the high infiltrative capacity of glioma cells. Tumor infiltration into the surrounding tissue impairs tumor removal but also impedes local tumor irradiation [22]. Several in vitro assays measured the radiosensitivity of glioma cell lines with the colony formation assay and all showed survival curves with the typical shoulder in the lower doses (1–2 Gy) and dropping survival fractions at higher doses (3–10 Gy). The dose that reduces the survival fraction to 50% (D50) was determined as ~2 Gy, whereas the D10 (dose at which only 10% of cells survive) was dependent on the cell lines, in a range between 5 and 8 Gy [15,25,26]. However, also several xenograft models examined the radiosensitivity of gliomas. Taghian et al. [27] demonstrated that the TCD50 (radiation dose that controls 50% of the tumors) in 10 different subcutaneous GBM xenograft tumor models varied between 32.5 and 75.2 Gy. Likewise, Baumann et al. [28] showed that five different subcutaneous glioma xenograft models exhibited a TCD50 between 73 and 120 Gy. Besides, Suit et al. [29] revealed that the radioresistance of the applied glioma models was higher than other tumor xenografts.

**Figure 1:**
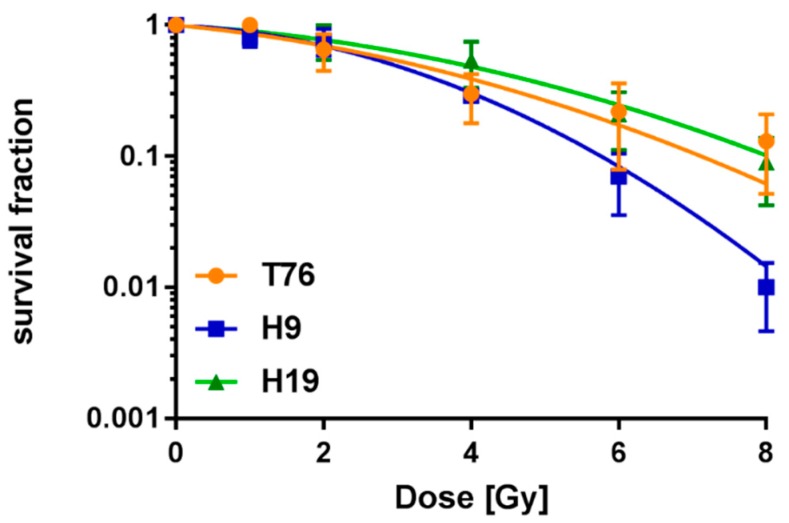
Survival fraction of three patient-derived glioma cell lines (T76, H9, and H19). Shown are three survival curves fitted with the linear-quadratic model after low linear energy transfer (LET) irradiation. Each survival fraction was calculated from three different measurement sets (*n* = 3), and the standard error of the mean (SEM) is shown.

We have also determined the survival fraction of three patient-derived glioma cell lines. As can be seen in Figure 1, all three measured cell lines show different D50 and D10 values after low LET irradiation (D50: T76: 3.2 Gy, H9: 2.9 Gy, H19 3.9 Gy; D10: T76: 7.1 Gy, H9: 5.8 Gy, H19: 8.0 Gy). Likewise, this finding might be caused by substantial heterogeneity in patient-specific tumor cell lines, causing different radiation sensitivity as, for example, T76 and H19 showed both isocitrate dehydrogenase (IDH) wildtype and O6-methylguanine-DNA methyltransferase (MGMT) was not methylated, whereas H9 was IDH mutated and MGMT was methylated.

### 2.2. Glioma Relapse Mostly Occurs Locally

Gliomas are characterized by a very aggressive growth pattern with diffuse infiltration. This feature greatly complicates glioma therapy. Aggressive glioma cells, exhibiting diffuse invasion, challenge the surgical resection as well as the targeted irradiation [30].

Whereas other aggressive tumors use the lymphatic and circulatory system to metastasize into organs, it was shown that the recurrence pattern of gliomas is mainly local and metastases are barely observed [22]. Ninety percent of GBMs have demonstrated to relapse in close proximity (1–2 cm) to the primary tumor, respective to the resection cavity [31,32]. Only 5–10% of all gliomas have been observed to relapse at more distant sites [33].

We have also determined the recurrence pattern of 178 glioma patients treated with reirradiation in our institution. As per the current literature, we could likewise show that most of the relapses occurred within the field of irradiation (see Table 1). From the viewpoint of the neuroradiologist, several imaging parameters can predict the prognosis as well as the recurrence pattern. Interestingly, postoperative infarct volume correlates with multifocal recurrence, recurrence with contact with the ventricle, and contact to dura [34]. This and other information can be used for personalized radiotherapy planning [35]; however, prospective validation remains open. Possibly, the migratory phenotype will correlate with progression patterns as well as growth models which then can be taken into account for target volume definition as well as dose individualization.

Recent findings suggest that this dismal prognosis is also due to early regression and progression after therapy. Tumor recurrence might be caused by chemo- and radioresistant cells. While the current treatment scheme only achieved moderately prolonged survival, the problem of local tumor recurrence has not yet been addressed [22]. However, as it is of utmost importance, there are several studies ongoing that are examining this finding and considering new therapeutic strategies [36].

### 2.3. Radiation-Enhanced Malignancy

To kill remaining cancer cells after surgery, almost every glioma patient undergoes photon irradiation as part of the multimodal treatment concept. It is generally known that glioma cells can migrate to distant sites and that those infiltrations limit patient outcome. Moreover, clinical irradiation has been shown to influence this migratory and invasive capacity. The extent to which irradiation influences the migratory and invasive phenotype is still controversially discussed. Whereas some preclinical studies demonstrate decreasing invasion or migration, other studies show increasing invasion or migration after photon radiation (see Table 2). There are even studies demonstrating that only some glioma cell lines exhibit radiation-enhanced malignancy.

**Figure 2:**
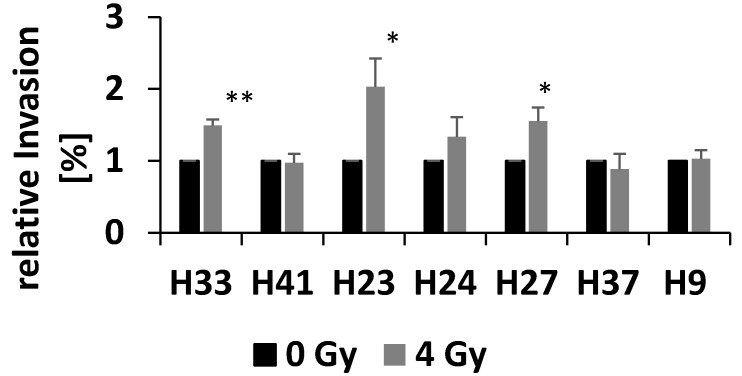
Invasion assay of primary patient-derived glioma cell lines. Shown are seven primary glioma cell lines that were examined for their invasion 24 h after 4 Gy X-ray irradiation. Each value represents the mean value of at least three biological replicates, and the SEM is depicted. Significance was calculated applying a Student’s *t*-test with * *p* ≤ 0.05 and ** *p* ≤ 0.01.

In addition to the data we published earlier this year, we can now show seven further primary patient-derived glioma cell lines that were examined for their invasion after low LET irradiation (see Figure 2). Again, we can conclude that only some primary glioma cell lines resulted in a radiation-enhanced invasion, whereas others showed no effect.

### 2.4. Toxicity Limits Dose Escalation

It is known that gliomas follow a clear dose–response relationship and that higher doses can be associated with higher survival [43,44]. Several studies have followed the concept of increasing the dose beyond 60 Gy, using either hyper- or hypofractionated concepts or sequential dose escalation [45,46,47,48,49]. Unfortunately, none of those studies have proven successful for clinical routine regarding a survival benefit. With the techniques available, however, toxicity increases with dose escalation [49]. With the continuous improvement in radiation precision, highly local dose escalation became possible, such as with stereotactic radiotherapy boost doses or with integrated boost concepts delivered by intensity-modulated radiotherapy (IMRT) [50,51,52]. Again, a survival benefit was not confirmed within a prospective randomized trial, although the studies do show a benefit for at least subgroups of patients.

The benefit of proton therapy regarding inverted dose profiles with sparing of healthy tissue has been exploited for local dose escalation. Fitzek et al. could clearly show that doses of 90 CGE (cobalt gray equivalent) correlate with increased survival; however, patients developed severe treatment-related toxicity requiring surgical intervention for necrosis [53]. Intraoperative dose escalation has been assessed without a substantial clinical benefit. However, with modern application possibilities such as Zeiss Intrabeam, using a sphere-shaped kV-application device, there is a convincing rationale for re-evaluation. Currently, intraoperative dose escalation is being evaluated in a prospective randomized trial [54].

Main limiting factors of percutaneous dose escalation regarding toxicity risk might be the missing relevant information for target volume definition. Identification of high-risk regions must discriminate in eloquent areas so they can be spared effectively. This might be possible with improved PET tracers or with radiomics and radiogenomics information derived from different imaging devices [55].

### 2.5. Molecular Biology

A considerable effort was put into the elucidation of underlying pathways and molecular changes that may explain the progress of the malignancy of gliomas after treatment, such as treatment resistance and radiation-enhanced malignancy. By analyzing those molecular changes, the aim is to develop targeted therapies, thereby improving patient outcome. To date, few molecular markers in glioma have reached real clinical relevance. One example, surely, is MGMT promotor methylation, which has been discussed especially in the context of alkylating agents such as temozolomide.

Also, molecular classifiers of outcome include loss of heterozygosity (LOH) of 1p19q, telomerase reverse transcriptase (TERT), alpha-thalassemia/mental retardation syndrome X-linked (ATRX), or isocitrate dehydrogenase (IDH)-1 or -2 mutations [56]. Many of those can predict outcome as they correlate with prognosis. However, none has led to a breakthrough in molecularly targeted treatments. Thus, it might be promising to identify molecular markers correlating with specific phenotypes, such as migration and invasion, and counteract with specific clinical approaches. In that context, specific targets have been in focus.

Glioma cell survival and proliferation caused by endothelial growth factor receptor (EGFR) overexpression have been shown several times [57]. Therefore, therapy concepts inhibiting EGFR, like gefitinib and erlotinib, have been examined [58]. However, those attempts failed in clinical trials [59,60]. By applying antiangiogenic agents like bevacizumab and cilengitide, further clinical trials examined other possibilities to inhibit growth-factor-mediated angiogenesis [61,62]. The development of blood vessels through angiogenesis was demonstrated to be essential for the formation of cellular disorders. By regulating proliferation and cellular movement, vascular endothelial growth factor (VEGF) showed to play a crucial role in angiogenesis [63]. Bevacizumab is a monoclonal antibody to VEGF. However, also integrins have been revealed to play an essential role in angiogenesis. Cilengitide is an integrin inhibitor, which blocks cell proliferation [64]. Both agents demonstrated impeded migration in clinical trials. However, despite promising results from phase I and II studies, both antiangiogenic agents revealed no statistically significant increase in overall survival [61]. As there are no validated biomarkers for antiangiogenic agents, recruitment for clinical trials was not performed on patient-specificity but more generally based on the MGMT status [65,66].

Furthermore, it was shown that irradiation promotes cytokine release as a result of radiation damage response. Therefore, cytokine secretion has been studied extensively [67]. The most important cytokines secreted by gliomas after irradiation were interleukins (IL-6, IL-8) and several growth factors (TGF-ß, VEGF) [68,69]. Another inflammation-associated pathway—cyclooxygenase-2 (COX-2) and its major enzymatic product prostaglandine E2 (PGE_2_)—has also been shown to contribute to the invasive and migratory phenotype of gliomas [70,71]. Furthermore, radiation can increase the production of pro-invasive PGE_2_ in human glioma cells [72]. Likewise, matrix metalloproteinases were extensively studied in connection with radiation-induced invasion [24,40,73]. Other molecular pathways that have been described to promote radiation-induced invasion in glioma include melanoma differentiation-associated gene 9 (MDA-9) [74], the transcription factor signal transducer and activator of transcription 3 (STAT3) [75], and the chemokine receptor CXCR4 [76].

## 3. Discussion

The naturally strong migratory and invasive capacity of glioma cells into healthy brain tissue is a serious and still barely understood component that causes poor prognosis of glioma patients. As the irradiation changes the tumor cell biology, numerous molecular alterations have been extensively studied but are still not fully understood. The activation of cell surface receptors and the overexpression of cytokines, growth factors, and proteases as well as signaling pathways have been analyzed. However, there is no precise mechanism found that could explain radiation-enhanced malignancy. This result already hints towards an interplay of multiple mechanisms that together promote treatment resistance. Tumor recurrence due to radio- and chemoresistance, as well as potentially radiation-enhanced malignancy, continues to challenge glioma therapy.

While several in vitro studies examined the migratory and invasive phenotype of glioma cells after irradiation, there are only limited in vivo clinical trials analyzing the effect of irradiation on the progression of gliomas.

Gliomas are known to show high treatment resistance (radio- as well as chemoresistance). Several in vitro studies proved D50 values of 2 Gy and D10 values between 5 and 8 Gy [27,28]. The diverging D10 already implicates the significant heterogeneity observed in gliomas. Of course, the sensitivity of in vitro colony formation assays from which D50 and D10 values are calculated does not match to patient outcome. This fact might be due to the optimal nutritional and oxygen conditions and the cell growth in 2D structures, without the tumor microenvironment [27]. However, the tumor heterogeneity within gliomas is already demonstrated. Likewise, several xenograft models showed this extensive radioresponse of diverse gliomas, again leading to the conclusion that different gliomas show a different radiation response.

The role of radiotherapy in cancer treatment is well known, as it has been used over decades to directly expose cells to cytotoxic effects to provide local tumor control. On the other hand, this local radiotherapy of primary tumors was shown to cause unpredictable effects on tumor malignancy [14,67]. As shown in Table 2, low LET irradiation modulates the migratory and invasive radiation response. Whether irradiation increases or decreases the migratory and invasive capacity depends on the cell line and on the applied radiation dose. However, the same cell line—U87MG—showed opposite effects in different research groups. Whereas Wild-Bode et al. demonstrated increased migration after 3 Gy irradiation [24], Goetze et al. observed decreased migration after irradiation with the same dose [20]. Therefore, diverging radiation responses can not only be due to different cell lines or doses but also to the institution. This finding might be explained by the fact that various assays were used to determine the migratory as well as invasive capacity (e.g., Boyden chamber, transwell assay, scratch assay), each with its standardizations from lab to lab. However, the fact that malignant gliomas are heterogeneous populations that underlie dynamic changes might also explain the contrary results [12]. Tumor cell communication and interaction between the healthy brain tissue and the tumor environment might be a factor in promoting this tumor heterogeneity. As observed by the different radiation responses, radiation-enhanced migration and invasion as well as tumor heterogeneity seems to play an important role. In a study published by us before [15], we could even show that primary patient-derived glioma cell lines showed different reaction after low LET irradiation. Our current study validates this finding. As primary cells are closer to the situation in the tumor, this demonstrates the importance of the heterogeneity of glioma cells. As shown in Table 2, only some (primary) glioma cell lines exhibited decreased migratory and invasive capacities, whereas others showed adverse effects. Several studies proved that many glioma cell lines showed radiation-enhanced migration and invasion [17,37,39]. However, to date, clinical studies are still missing. Our data have shown that patient-specific invasion is heterogeneous, and radiotherapy as a trigger can act in different intensities, even with comparable doses [15]. Therefore, personalized concepts taking into account the migratory and invasive potential regarding radiation dose or target volume modification or the addition of molecularly targeted agents inhibiting tumor cell migration and invasion could be applied in the future. However, these treatments should be individualized to the patient- and tumor-specific properties.

Today almost all glioma patients are treated with photon irradiation, and therefore, radiation-enhanced migration or invasion might play an essential role in patient survival. Considering future glioma therapy, a more personalized treatment concept should be essential. Being able to differentiate between patients benefiting from photon irradiation and those showing adverse effects, limiting their therapy success, might help to improve patient outcome.

This article suggests that irradiation of glioma cells modulates the migratory and invasive phenotype. Depending on the assay and dose applied, diverging results were observed. However, tumor heterogeneity seems to play a decisive role. In recent studies, low LET irradiation showed increasing as well as decreasing effects on glioma migration and invasion. Hence, this investigation emphasizes the importance of personalized medicine, even though there is a considerable need for further clinical studies validating the results from in vitro assays.

## 4. Materials and Methods

### 4.1. Primary Cell Isolation from Patient-Derived Tumor Tissue

Patient-derived primary cells were isolated as described before [15]. In brief, tumor tissue was mechanically and enzymatically digested at room temperature before being plated on GelTrex-coated plastic T25 flasks and cultivated in RPMI 1640 (R8758, Sigma, Taufkirchen, Germany) supplemented with 10% FCS (F7524, Sigma) and 1% penicillin/streptomycin (P0781, Sigma). Patients ≥ 18 years of age with primary glioma tumors were eligible for participation in the experimental RadGlio study. Exclusion criteria were pregnancy and breastfeeding, missing consent, age below 18 years, and patients that were not eligible for surgery. Patients’ characteristics are shown in Table 3. All subjects gave their informed consent for inclusion before they participated in the study. The study was conducted in accordance with the Declaration of Helsinki, and the protocol was approved by the Ethics Committee of the Technical University of Munich (TUM) (394/16S).

### 4.2. Low LET Irradiation

Low LET X-ray irradiation was performed using a clinical linear accelerator (ONCOR, Siemens, München, Germany) at a dose rate of 3 Gy/min (6 MV photons with 2 cm water-equivalent build-up).

### 4.3. Colony Formation Assay (CFA)

Cells were irradiated with different doses (0, 1, 2, 4, 6, and 8 Gy). Three T25 flasks were irradiated per dose. After irradiation, cells were trypsinized and cells from each flask were plated into several 12-well plates. Twelve days after plating, the colonies were fixed with ice-cold methanol, stained with 0.1% crystal violet, and counted. Colonies consisting of more than 50 single cells were counted as one colony with the GelCount™ (Oxford Optronics, Abingdon, UK). Survival curves were fitted according to the linear-quadratic model (LQ) with the equation

ln SF = −α × D − β × D^2^

by the GraphPad Software. Each CFA was performed three times.

### 4.4. Invasion Assay

Immediately after irradiation, the cell culture medium was exchanged with medium containing 0.5% FCS (serum starvation). Twenty-four hours after irradiation, 2 × 10^4^ cells were seeded per insert in medium containing 0.5% FCS. Corning 24-well, 8-µm pore sized transwell inserts, uncoated, as well as coated with matrigel, were used according to manufacturer’s instructions. Twenty-four hours after seeding, noninvading cells were removed, and invaded cells were fixed with ice-cold methanol and stained with 0.1% crystal violet. Five independent microscope fields were counted using a Zeiss Imager Z1m microscope at 10× magnification. All invasion assays were performed at least in triplicate. The invasion was calculated as the mean number of cells invading through the matrigel-coated membrane divided by the mean number of cells migrating through the uncoated membrane (control insert). Relative invasion of irradiated cells was normalized to sham-irradiated cells.

### 4.5. Statistical Analysis

Mean values were calculated and are presented as ±standard error of the mean (SEM). Significance was evaluated by the Student’s *t*-test (GraphPad Prism, La Jolla, CA, USA). A *p*-value of ≤ 0.05 was considered as statistically significant.

### 4.6. Literature Search Strategy

To identify relevant literature, a PubMed search was performed using the following key words: radiation, glioma, glioblastoma, invasion, and migration.

## 5. Conclusions

This study discussed that radio- and chemoresistance, as well as the different findings of radiation-enhanced migration and invasion of gliomas, could be accredited to high inter- and intratumor heterogeneity. We conclude that the role of tumor heterogeneity should not be neglected concerning glioma therapy. Furthermore, we suggest that there is a clear need for optimal, patient-specific irradiation. However, other radiation qualities, such as high LET irradiation, have already been suggested to improve glioma treatment.

## Figures and Tables

**Table 1:** List of recurrence patterns from glioma patients treated with radiotherapy at the Technical University of Munich (TUM). Studies were carried out independently by the Department of Radiation Oncology (RO) and the Department of Neuroradiology (NR).

WHO Grade [Department]	*n*	Field Border		Infield		Outfield	
WHO III [RO]	13	0	0.0%	12	92.3%	1	7.7%
WHO IV [RO]	80	9	11.3%	68	85.0%	3	3.8%
WHO IV [NR] [34]	85			72	84.7%	13	15.3%

**Table 2:** In vitro studies investigating migration and invasion after low LET irradiation. Not determined values are marked with n.d.

Author	Cell Line	Dose	Migration	Invasion
Bagida et al. [37]	U251, U87	8 Gy	Increasing tendency	Increasing tendency
Cordes et al. [38]	A172, U138, LN-229, and LN-18	6 Gy	n.d.	A172: decreased, U138, LN229, and LN18: no effect
De Bacco et al. [39]	U251	10 Gy	Increased	Increased
Fehlauer et al. [19]	GaMg and U87MG	5 Gy (U87) 10 Gy (GaMg)	Decreased	n.d.
Goetze et al. [20]	U87MG	1, 3, and 10 Gy	1 Gy: no effect 3 and 10 Gy: decreased	n.d.
Nalla et al. [17]	DAOY, D283	7 Gy	Increased	Increased
Park et al. [40]	U251, U373, LN-18, and LN428	1, 2, 3, and 5 Gy	n.d.	U251 and U373: increased, LN18 and LN428: no effect
Rieken et al. [41]	U87MG, LN-229	2 and 10 Gy	2 Gy: increased, 10 Gy: no effect	n.d.
Steinle et al. [16]	T98G, U87 MG	2 Gy	Increased	n.d.
Wank et al. [15]	LN-229, LN-18, U87 MG, and 7 primary cell lines	4 Gy	n.d.	LN229 and U87: increased, LN18: no effect; 4 primary cell lines: increased 2 primary cell lines: increasing tendency 1 primary cell line: no effect
Wild-Bode et al. [24]	U87MG, LN-229, and LN18	1, 3, and 6 Gy	No effect after 1 Gy but increased after 3 and 6 Gy	1 Gy: no effect 3 and 6 Gy: increased
Zhai et al. [42]	Two primary glioma cell lines	2, 4, 6, and 8 Gy	Increased	Increased
Zheng et al. [21]	A172, LN-229	5 Gy	Decreased	Decreased

**Table 3:** Patients’ characteristics. MGMT: O6-methylguanin-DNA methyltransferase, IDH: isocitrate dehydrogenase.

Patients’ Characteristics	Groups	All	All (%)
**Sex (No.)**			
	Male	4	44.4
	Female	5	55.6
**Age (years)**			
	Median	45	
	Range	24–89	
**MGMT-Status (No.)**			
	unmethylated (<8%)	4	44.45
	methylated (≥8%)	4	44.45
	unknown	1	11.1
**IDH Status (No.)**			
	wildtype	5	55.6
	mutated	4	44.4

## Abbreviations

ATRX	Alpha-thalassemia/mental retardation syndrome X-linked
CGE	Cobalt gray equivalent
CNS	Central nervous system
D10	Dose where only 10% survive
D50	Dose where only 50% survive
EGFR	Endothelial growth factor receptor
GBM	Glioblastoma multiforme
IDH	Isocitrate dehydrogenase
IMRT	Intensity-modulated radiotherapy
LET	Linear energy transfer
LOH	Loss of heterozygosity
MGMT	O6-methylguanine-DNA methyltransferase
SEM	Standard error of the mean
TCD50	Radiation dose that controls 50% of the tumors
TERT	Telomerase reverse transcriptase
TGF	Transforming growth factor
VEGF	Vascular endothelial growth factor
WHO	World Health Organization
